# The Role of Chemokines in Breast Cancer Pathology and Its Possible Use as Therapeutic Targets

**DOI:** 10.1155/2014/849720

**Published:** 2014-08-05

**Authors:** M. Isabel Palacios-Arreola, Karen E. Nava-Castro, Julieta I. Castro, Eduardo García-Zepeda, Julio C. Carrero, Jorge Morales-Montor

**Affiliations:** ^1^Departamento de Inmunología, Instituto de Investigaciones Biomédicas, Universidad Nacional Autónoma de México, A.P. 70228, 04510 México, DF, Mexico; ^2^Dirección de Enfermedades Crónicas y Cáncer, Centro de Investigación Sobre Enfermedades Infecciosas, Instituto Nacional de Salud Pública, 62115 Cuernavaca, MOR, Mexico

## Abstract

Chemokines are small proteins that primarily regulate the traffic of leukocytes under homeostatic conditions and during specific immune responses. The chemokine-chemokine receptor system comprises almost 50 chemokines and approximately 20 chemokine receptors; thus, there is no unique ligand for each receptor and the binding of different chemokines to the same receptor might have disparate effects. Complicating the system further, these effects depend on the cellular milieu. In cancer, although chemokines are associated primarily with the generation of a protumoral microenvironment and organ-directed metastasis, they also mediate other phenomena related to disease progression, such as angiogenesis and even chemoresistance. Therefore, the chemokine system is becoming a target in cancer therapeutics. We review the emerging data and correlations between chemokines/chemokine receptors and breast cancer, their implications in cancer progression, and possible therapeutic strategies that exploit the chemokine system.

## 1. Introduction

Chemokines are small proteins that primarily regulate the trafficking of leukocytes under homeostatic conditions and during specific immune responses. They share a secondary structure, and based on their amino acid composition—specifically, the presence of a conserved tetra-cysteine motif—they are grouped into 4 families: C, CC, CXC, and CX3C [1, 2]. Chemokines guide the migration and adhesion of leukocytes and influence other cellular functions, such as proliferation, maturation, angiogenesis, and malignant transformation [1, 2]. These effects are mediated by binding to G-protein-coupled receptors (GPCRs) with 7 transmembrane domains [2].

The chemokine system comprises almost 50 chemokines and approximately 20 chemokine receptors [2, 3]. Upon the binding of different chemokines to the same receptor, there can be a variety of biological effects. Complicating the system further, the effects also depend on the cellular microenvironment.

Breast cancer has gained particular relevance in recent years due to the high incidence in both developed and less developed regions [4]. Breast cancer is basically defined by the presence of a malignant tumor that originates from breast tissue, either from lobes, ducts, or stroma. The tumor cells proliferate and are able to invade surrounding tissues, lymph nodes and distant organs. According to the size of the primary tumor, the involvement of lymph nodes, and the presence of distant metastasis, the stage of breast cancer can be determined, ranging from stage 0 to stage IV [5].

The intervention of the immune system in cancer does not begin with the fighting and effort to restrain an established tumoral mass but with the detection of transformed cells since they began proliferating. In the late 50's, Burnet proposed the* immunosurveillance* theory [6], which proposes that the immune system cells are capable of detecting transformed cells, attacking them and eliciting an specific (adaptive) response to eventually succeed and eliminate them all or fail, leading then to the formation of a tumoral mass and cancer onset.

Nowadays, it is known that this process is more complex than previously thought and consists not only of that simple event series, but can comprise alternative processes, such as* immunoediting *and even* immunosubversion *[7, 8]. The previous could be depicted by the paradoxical fact that a greater infiltration of immune cells in breast cancer neoplasia has been correlated with a worse disease prognostic, and how this can also be explained by the polarization phenomena that immune cells experience in the tumoral microenvironment, which induces the acquisition of a protumoral phenotype.

Chemokines and chemokine receptors play a key role along these processes, since they not only comprise the main regulatory system leading leukocyte infiltration in primary tumors, but also intervene in cancer cells proliferation and in metastasis guidance.

Nearly every tissue expresses chemokines and chemokine receptors. Normal breast expresses a set of chemokines at generally low levels [9]. We review the emerging data and correlations between chemokines and breast cancer, from their implications in cancer progression to therapeutic strategies that exploit the chemokine system.

## 2. Breast Cancer Cells Proliferation and Tumor Growth: Is There a Function for Chemokines?

Chemokines not only are associated with the establishment of a protumoral microenvironment and organ-directed metastasis, but also mediate disease progression, favoring the growth and proliferation of tumor cells. Several chemokines have been described as participating in these processes.

One of those chemokines implicated in breast cancer progression is CCL2 (formerly known as MCP-1), which is a potent chemotactic factor that regulates the migration and infiltration of monocytes, memory T lymphocytes, and NK cells, signaling through CCR2 and CCR4 [10]. CCL2 is expressed at high levels in both tumor and tumor microenvironment cells, exerting its protumoral effects indirectly by promoting angiogenesis and enriching leukocyte infiltration [11, 12], primarily with tumor-associated macrophages (TAMs), which produce immunomodulatory factors that promote angiogenesis and tumor growth.

Moreover, CCL2 mediates development of the cancer stem cell (CSC) phenotype. Cancer-associated fibroblasts (CAFs) and fibroblasts that are activated by coculture with cancer cells secrete high levels of CCL2, which affects the sphere-forming phenotype (stem cell-specific) of breast cancer cells and CSC self-renewal [13]. The promotion or CSC phenotype is of great relevance in cancer biology, given that this population of self-renewing, chemo- and radioresistant cells is thought to maintain the tumor heterogeneity, as well as giving rise to metastasis.

Another member of the CC family of chemokines that has been related to breast cancer progression is CCL20. This chemokine primarily targets lymphocytes and dendritic cells, although it also attracts neutrophils weakly, upon binding to receptor CCR6. Recently, Marsigliante and colleagues [14] correlated high CCL20 concentrations with extensive cellular proliferation, mediated by increased cyclin E (which is required for the transition from G1 to S phase) and decreased p27 (an inhibitor of cyclin D).

Cell cycle regulation is clearly a key element in cancer progression and recently CXCL8, a member of the CXC family, has been identified as a promoter of cell cycle progression. Shao and colleagues demonstrated that silencing CXCL8 using siRNA in the MDA-MB-231 breast cancer cell line resulted in the upregulation of p27, downregulation of cyclin D1, and thus a delay in the progression from G1 to S phase [15]. Besides cell cycle regulation, CXCL8 has been implicated in CSC phenotype. It has been reported that breast cancer stem cells express CXCR1, which upon binding of CXCL8 increase their activity (measured as sphere-formation) and self-renewal [16].

Although traditionally implicated in organ-directed metastasis, CXCR4 is a chemokine receptor that has been linked to cancer progression. Its constitutive activation in MCF-7 breast cancer cells enhances tumor growth and metastasis, which can be reversed by its inhibition [17].

In contrast, the chemokine CXCL14, which is abundantly expressed in normal tissue but downregulated in breast cancer tissue and cell lines, negatively regulates the growth and metastasis of breast cancer as its expression is positively associated with patient survival and a lower incidence of metastasis. Overexpression of CXCL14 was recently reported to inhibit cell proliferation* in vitro* and decrease xenograft tumor growth* in vivo *[18].

## 3. Chemokines and Tumor Microenvironment

Cancer cells are not the only decisive factor in the course of the disease—there are other factors, both systemic and in the tumor microenvironment, that can limit or promote the growth of cancer cells and their mobility and dissemination to other organs.

**Figure 1:**
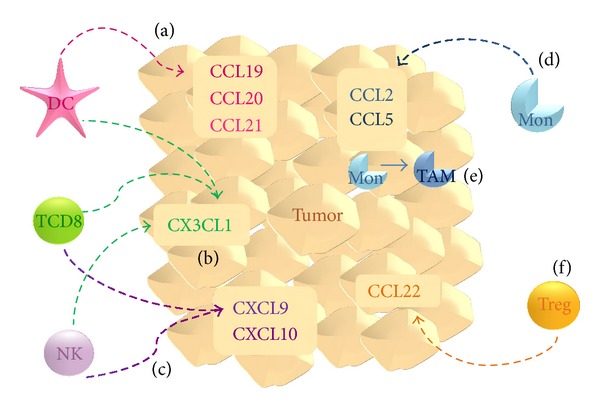
Chemokines influx the tumor microenvironment. (a) CCL19, CCL20, and CCL21 act as chemoattractants for dendritic cells (DC); (b) CX3CL1 has been related to infiltration of DCs as well as activated (cytotoxic) CD8+ T lymphocytes and NK cells. These last two populations are also chemoattracted by (c) CXCL9 and CXCL10. DCs, T CD8+ lymphocytes, and NK cells are thought to contribute to antitumoral immune response. (d) CCL2 and CCL5 are both chemoattractants for monocytes (Mon), which (e) within tumor microenvironment acquire a TAM phenotype. (f) CCL22 expression correlates with T_reg_ infiltration, which together with TAMs promotes tumor survival and progression.

When talking about the progression of cancer, one of the key elements of the tumor microenvironment is the myeloid cell population, particularly macrophages. On recruitment to the tumor microenvironment, macrophages are influenced by the cytokine milieu and local growth factors, resulting in the acquisition of a protumoral phenotype. The resulting TAMs produce angiogenic and immunomodulatory factors (e.g., IL-10 and TGF-*β*) which induce regulatory T lymphocytes (T_reg_) and facilitate remodeling of the extracellular matrix, promoting cancer cell motility. Because a primary function of chemokines is to attract and direct leukocytes (Figure 1), their significance is evident, regarding leukocyte infiltration into the tumor microenvironment.

CCL2 and CCL5 (RANTES) chemokines have been extensively studied in breast cancer. As mentioned before, CCL2 is a potent chemoattractant of monocytes (Figure 1); in addition to monocytes, CCL5 recruits T helper type 2 lymphocytes (Th2) and eosinophils, signaling through the receptors CCR1, CCR3, and CCR5 [2, 11]. These chemokines have similar expression patterns—they are detected in cancer cells in primary tumors, tumor-infiltrated lymph nodes, distant metastases, and cells that are adjacent to the tumor (e.g., TAMs and fibroblasts) but are expressed at low levels in healthy breast tissue [11, 12].

An increase in the CCL2 expression levels correlate with the extent of TAM infiltration in primary tumors and in animal xenograft models, a causal relationship based on findings where blocking CCL2 with neutralizing antibodies decreases macrophage infiltration, tumor growth, and angiogenesis (associated to some extent with the presence of TAMs) in a mouse model of breast cancer [19, 20]. Although CCL5 is also linked to macrophage infiltration in animal models of breast cancer [21, 22], in human breast xenografts, CCL5 expression correlates negatively with macrophage recruitment [19], implying the participation of other chemokines.

In addition to macrophages, the leukocyte infiltrate in the tumoral niche includes T lymphocytes, dendritic cells (DCs), NK cells, and other granulocytes, which also influence the fate of cancer cells at the cellular level and by modifying the tumoral microenvironment with cytokines and chemokines. The chemokines CCL19, CCL20, and CCL21 regulate the traffic of DCs (Figure 1), and their overexpression in experimental tumor systems has antitumoral effects [12]. Although the function of DCs in cancer immunology is still under investigation, the effects of the overexpression of these chemokines correlate with increased DC infiltration.

T lymphocytes and NK cells are important populations in tumor immunology—activated lymphocytes orchestrate immune response against cancer cells, and NK cells are central innate effectors that recognize and have cytotoxic effects on stressed and transformed cells. In this context, CXC chemokines CXCL9 (also known as Mig) and CXCL10 (IP-10) control the migration of activated T cells and NK cells [23, 24], which can enhance the antitumoral response (Figure 1). Moreover, these chemokines belong to the antiangiogenic ELR(−) CXC subfamily (further discussed later) [25, 26]. In addition, CXCL12 (SDF-1) was recently shown to promote CD8^+^ cytotoxic T lymphocyte activity when overexpressed in a syngeneic model of breast carcinoma [27].

CX3CL1 (also known as Fractalkine) is the only known member of the CX3C chemokine family and signals through CX3CR1. This receptor is expressed on monocytes, NK cells, and T lymphocytes, to mediate several functions, including migration, adhesion, and proliferation [28]. As discussed, T lymphocytes and NK cells are key populations in antitumoral immunity—a response in which CX3CL1 is thus expected to be involved. Park and colleagues [29] described a positive correlation between CX3CL1 expression in breast carcinoma specimens and the number of stromal T CD8^+^ lymphocytes, intratumoral DCs, and stromal NK cells (Figure 1). Consistent with these results and the antitumoral properties of these subpopulations, elevated CX3CL1 expression may be associated with significantly better disease-free survival.

Conversely, T_reg_ infiltration is associated with a poor prognosis. It is now clear that T_regs_ induce an immunomodulatory state by producing IL-10 and TGF-*β*, which inhibit APC maturation and the expression of costimulatory molecules as well as decreasing the cytotoxic potential of NK cells and cytotoxic T lymphocytes. CCL22 is a chemokine that signals through CCR4 and is a chemoattractant for monocytes, dendritic cells, NK cells, and chronically activated T lymphocytes. Recently, CCL22 was linked to T_reg_ infiltration (Figure 1) in gastric, esophageal, and ovarian carcinomas [30–32]. Consistent with its significance in T_reg_ infiltration, breast tumors that lack CCL22 are not infiltrated by the T_reg_ subpopulation [33]. Moreover, in human breast carcinoma cell lines, CCL22 was secreted at low basal levels and upregulated in response to inflammatory signals.

## 4. The Function of Chemokines in Angiogenesis

Angiogenesis is a hallmark of cancer. The resulting tumor-associated neovasculature that is generated addresses the tumor's growing demands for nutrients and oxygen [34] and enables the tumor to grow and avoid excessive necrosis.

**Figure 2:**
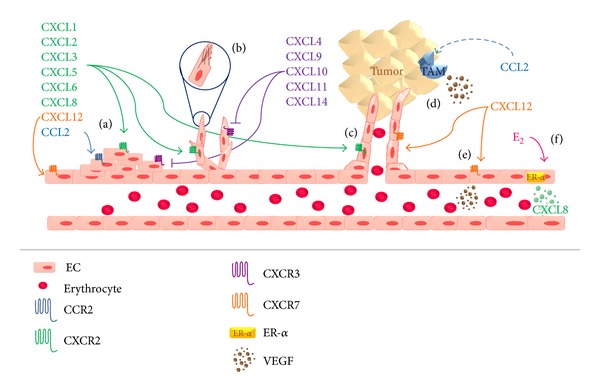
Chemokines involvement in angiogenesis. Angiogenic ELR^+^ chemokines act through CXCR2 receptor to promote (a) proliferation of endothelial cells (EC), (b) stress fibre assembly, and (c) tube formation. On its behalf, antiangiogenic non-ELR^+^ chemokines, via CXCR3, inhibit these processes. CCL2 also promotes angiogenesis via CCR2, (a) stimulating EC proliferation and (d) in an indirect manner by increasing TAM infiltration, which secrete angiogenic factors like VEGF. CXCL12 acts through CXCR7 to promote (a) EC proliferation and (e) VEGF production by these cells. It has been reported that (f) estrogen (E_2_) stimulates EC secretion of CXCL8.

The CXC chemokine family comprises angiogenic and antiangiogenic chemokines (Table 1). Angiogenic chemokines, such as CXCL1, CXCL2, CXCL3, CXCL5, CXCL6, and CXCL8, are generally distinguished by an ELR motif. These chemokines mediate their angiogenicity through CXCR2 and interact alone or with other angiogenic factors (e.g., VEGF) to effect angiogenesis (Figure 2) [26]. CXCL8 is considered one of the most potent inducers of angiogenic processes [12] such as rapid stress fiber assembly, chemotaxis, enhanced proliferation of and tube formation by endothelial cells [26]. Noteworthy, Haim and coworkers [35] reported that estrogen upregulates the transcription and secretion of CXCL8 in breast tumor cells additively through estrogen receptor *α* (ER*α*), adding a novel role of estrogen in promoting tumor growth (Figure 2).

Although CXCL12 is a non-ELR-CXC chemokine, it has been implicated as an angiogenic chemokine based on evidence of its involvement in blood vessel formation, inducing endothelial cell migration and proliferation, stimulating tube formation, and enhancing VEGF release (Figure 2) [12].

Angiostatic CXC chemokine family members include CXCL4, CXCL9, CXCL10, CXCL11, and CXCL14. CXCL4, CXCL9, and CXCL10 signal through CXCR3, which, on ligand engagement, blocks microvascular endothelial cell migration and proliferation (Figure 2) in response to various angiogenic factors [26].

CCL2 and CCL5 have been also suggested to shift the balance in the tumor microenvironment towards increased vascularity. CCL2 acts directly on endothelial cells to promote angiogenesis and correlates closely with positive endothelial growth regulators, such as vascular endothelial growth factor (VEGF), thymidine phosphorylase (TP), and CXCL8 [20]. In an indirect manner, CCL2 increases the presence of TAMs, which produce other angiogenic factors [11] (Figure 2).

In addition to the development of new blood vessels, tumors also undergo a process denominated Lymphangiogenesis—the growth of lymphatic vessels—which contributes to lymphatic metastasis [36] and is thus a major event in the development and spread of cancer. Peritumoral lymphangiogenesis involves the secretion of VEGF-C and VEGF-D, which act on the lymphatic endothelium and are upregulated in the MCF10 breast cancer cell line [37]. Further, CXCL12 is a chemoattractant for lymphangiogenic endothelial cells (LECs), inducing the migration and tubule formation of LECs* in vitro* and lymphangiogenesis* in vivo* and correlating with lymphatic vessel density in cancer tissues [36].

## 5. The Function of Chemokines in Metastasis

Metastasis is the dissemination of cancer cells to distant organs and tissues, such as the liver, lung, brain, and bone. This process is the most devastating attribute of cancer and significantly influences its morbidity and mortality [38]. Cancer metastasis is not a fortuitous or randomly driven process but is governed by many factors that, for example, allow cancer cells to move, detach from the ECM (which is achieved by the expression of matrix metalloproteases and heparanase), intravasate, migrate to distant organs and be able to fluorish in a different niche from the one in which they developed. How other cellular populations in the tumor microenvironment contribute to ECM remodeling is beyond the scope of this section, but notably cancer-associated fibroblasts (CAFs) have a significant function in this process.

The CXCL12-CXCR4 axis is one of the most extensively studied pairs in metastasis, primarily with regard to its involvement in organ-directed metastasis. Its function in metastasis begins with cancer cell mobility—the binding of CXCL12 to CXCR4 activates various intracellular signal transduction pathways and effector molecules that regulate chemotaxis, migration, and adhesion. Low-CXCR4-expressing MCF-7 cells fail to metastasize when injected into mice, whereas CXCR4-high MDA-231 cells are efficient in forming distant organ metastases [39]. Similarly, CCL21, through its receptor CCR7, triggers actin polymerization, pseudopodia formation, and the directional migration and invasion of breast cancer cells, particularly to lymph nodes, where CCL21 is highly expressed [40].

CXCR4 expression is higher in malignant breast tumor compared with its normal counterpart [40]. It controls chemotaxis toward its ligand, CXCL12, which is highly expressed in the lung, bone, liver, and lymph nodes, organs to which breast cancer cells preferentially metastasizes [41, 42]. With regard to brain metastasis, it remains unknown how cancer cells breach the brain-blood barrier (BBB) and invade this tissue, but it is possible that CXCL12-expressing CNS cells are chemoattractants for metastatic breast cancer cells [43].

Triple-negative breast cancer (TNBC) is a very aggressive subtype with few therapeutic alternatives and a poor prognosis. CXCR4 expression was recently reported to be more frequent in TNBC versus other subtypes, and CXCR4-positive patients had a significantly higher rate of metastasis, larger primary tumors, and shorter overall- and disease-free survival [44].

Chemokines that are expressed by osteoblasts and bone marrow endothelial cells have been implicated in driving bone metastasis. During their differentiation into osteoblasts, mesenchymal stem cells secrete CCL2, which is believed to mediate the migration of cancer cells, a process that is partially inhibited by anti-CCL2 [45]. CX3CL1 is expressed in a membrane-bound form and is exposed to the luminal side of human bone marrow endothelial cells, whereas its receptor, CX3CR1, is expressed in normal and malignant mammary glands [46]. Breast cancer cells that express high levels of CX3CR1 have a greater propensity toward bone metastasis; consistent with these data, studies in CX3CL1-null transgenic mice suggest that the absence of this chemokine impairs the dissemination of cancer cells to bone [46].

Notably, Hernandez et al. [47] reported that the binding of CXCL12 to CXCR4 and CXCR7 elicits disparate cellular responses. CXCR4 controls chemotactic and invasive behavior (*in vivo* motility and intravasation) in response to CXCL12, whereas CXCR7 enhances primary tumor growth and angiogenesis but decreases* in vivo* invasion, intravasation, and metastasis formation.

As discussed, CCL2 mediates TAM infiltration and generates an amplification loop, upregulating CCL2 in TAMs, which is associated with the expression of membrane type 1-matrix metalloprotease (MT1-MMP) [11]. Similarly, other chemokines and receptors, such as CCL5, CCL20 (via CCR6), CXCL12, and CXCR7, induce or increase the expression of MMPs [12, 14, 47]. CXCL7 has been reported to be linked to greater heparanase activity in MCF-7 breast cancer cells [37].

Recently, Chen and colleagues [48] linked TAM-produced CCL18 with cancer cell invasiveness and identified PITPNM3 (a membrane-associated phosphatidylinositol transfer protein) as its receptor. In this report, CCL18 colocalized with CD68 (a TAM marker) in most invasive breast carcinoma samples. In* in vitro* experiments, the group showed that the invasiveness of MDA-MB-231 cells and primary breast cancer cells was enhanced equally by the addition of recombinant CCL18 and coculture with TAMs, whereas the addition of anti-CCL18 and CCL18-siRNAs reduced the number of invasive cancer cells. Based on these findings, it is concluded that TAMs are a source of CCL18 and there may be a close relationship between CCL18 and invasiveness.

## 6. Therapeutic Targeting of Chemokines in Breast Cancer

Chemoresistance is a significant obstacle in cancer treatment, because cancer cell subpopulations that survive chemotherapy can proliferate and reemerge as a more aggressive variant, limiting subsequent therapeutic options. CCL25, via CCR9, confers a survival advantage to breast cancer cells by inhibiting cisplatin-induced apoptosis in a PI3K-dependent manner, in addition to activating cell survival signals through Akt [49]. Acharyya et al. reported a notable feedback mechanism between chemotherapy treatment and chemokine-induced chemoresistance [50], in which CXCL1 and CXCL2 attract CD11b^+^GR1^+^ myeloid cells, which produce other chemokines, including S100A8/9, that enhance cancer cell survival. Although chemotherapeutic agents kill cancer cells, they induce TNF-*α* production by endothelial and stromal cells, which upregulates CXCL1 and CXCL2 in cancer cells, amplifying the CXCL1/2-S100A8/9 loop and affecting chemoresistance.

Chemokines and chemokine receptors are appealing targets for cancer treatment, based on the wide range of processes that they influence. For instance, chemokine receptors mediate critical steps in the development and spread of cancer, for which antagonists have been designed to inhibit signal transduction and impede the undesired effects of chemokines. In CXCL1- and CXCL2-induced chemoresistance, CXCR2 blockers have been shown to break the chemokine-chemoresistance cycle described above, augmenting the efficacy of chemotherapy [50].

As described, CCL5 mediates cancer cell invasiveness and signals through CCR5. CCR5 antagonists slow the invasion of basal breast carcinoma cells* in vitro* and decrease pulmonary metastasis in a preclinical mouse model of breast cancer, suggesting that CCR5 antagonists can be used as adjuvant therapy in patients with this breast cancer subtype [51].

Concerning the chemoresistant CSC population, it is known that chemotherapy induces the expression of CXCL8 in injured cells, which increases the activity and self-renewal of the former. Thus, the blockade of CXCL8 receptors CXCR1 and CXCR2 arises as a promising side-therapy attempting to avoid tumor recurrence [16, 52]. Furthermore, not only the number of CSCs but also a reduction in general tumor cell viability is achieved by the use of CXCR1 inhibitors [52].

Because the CXCL12-CXCR4 axis has significant function in breast cancer metastasis, it has also been targeted using CXCR4 antagonists. Williams and colleagues [27] reported that a CXCL12 analog with antagonist activity (CXCL12(P2G)) significantly inhibited metastasis in a syngeneic mouse model of breast carcinoma. Conversely, T140 analogs are peptidic CXCR4 antagonists, originally developed as anti-HIV agents that inhibit CXCL12-induced migration of MDA-MB-231 breast cancer cells* in vitro* and mitigate pulmonary metastasis* in vivo *[53].

Chemokines that promote and enhance the activity and interaction of immune cells have been exploited as a prophylactic approach. CCL19 and CCL21 regulate the encounter between DCs and T lymphocytes in lymph nodes, for which they can be considered as important natural adjuvants for immune response [54, 55]. These chemokines have been used in DNA vaccines, amplifying their immunogenicity, inducing a Th1-polarized immune response, and substantially improving their protective effects in BALB/c mice [55]. Oncolytic viruses are also promising cancer treatments; this virotherapy, in combination with a CXCR4 antagonist, has increased efficacy over oncolysis alone in a triple-negative breast carcinoma syngeneic mouse model; systemic delivery of the armed virus after resection of the primary tumor inhibits the development of metastasis and increases overall tumor-free survival [56].

## 7. Concluding Remarks

Rapidly accumulating data in breast cancer immunology from recent years suggest that many established and widely accepted paradigms should be revised. In breast cancer, whereas chemokines are primarily associated with the generation of a protumoral microenvironment and organ-directed metastasis, they also appear to mediate disease progression, favoring the growth and proliferation of tumor cells.

Recent studies suggest that inhibiting local chemokines signaling in the tumor by blocking particular receptors or using analogs with antagonist activity could be a new rationale promising strategy for controlling tumor development and progression. Thus, the development of drugs that specifically target the chemokine axis will be invaluable in the treatment of breast cancer, in which inflammation has a major role.

## Figures and Tables

**Table 1:** Angiogenic and antiangiogenic members of CXC chemokine family.

Angiogenic ELR^+^ chemokines	Antiangiogenic non-ELR^+^ chemokines
CXCL1	CXCL4
CXCL2	CXCL9
CXCL3	CXCL10
CXCL5	CXCL11
CXCL6	CXCL14
CXCL7	
CXCL8	
